# Genetically Modified Mouse Models for Sarcoma Research: A Comprehensive Review

**DOI:** 10.1007/s11912-025-01717-8

**Published:** 2025-10-22

**Authors:** Piotr Remiszewski, Eryk Siedlecki, Marlena Wełniak-Kamińska, Michał Mikula, Anna M. Czarnecka

**Affiliations:** 1 Department of Soft Tissue/Bone Sarcoma and Melanoma, Maria Sklodowska- Curie National Research Institute of Oncology, Warsaw, Poland; 2 Medical Faculty, Medical University of Warsaw, Warsaw, Poland; 3 Small Animal Magnetic Resonance Imaging Laboratory, Mossakowski Medical Research Institute, Polish Academy of Sciences, Warsaw, Poland; 4 Department of Genetics, Maria Sklodowska-Curie National Research Institute of Oncology, Warsaw, 02-781 Poland; 5 Department of Experimental Pharmacology, Mossakowski Medical Research Institute, Polish Academy of Sciences, Warsaw, Poland; 6 Outpatient Chemotherapy Department, Maria Sklodowska-Curie National Research Institute of Oncology, Roentgena 5 St, Warsaw, 02-781 Poland

**Keywords:** Soft tissue sarcoma, Genetically engineered mouse, CRISPR-Cas9, Cre-loxP, Animal models, Preclinical model

## Abstract

**Purpose of Review:**

Sarcomas are a heterogeneous group of over 170 malignant tumours of mesenchymal origin. The poor prognosis highlights the need for novel therapeutic strategies. Preclinical modelling is essential, yet challenging, given that sarcomas differ substantially from carcinomas and resources are very limited.

**Recent Findings:**

GEMMs allow for the precise modelling of recurrent sarcoma genetics. The Cre-loxP system offer spatial and temporal control over the activation of oncogenes or the loss of tumour suppressors, while the CRISPR-Cas9 system enables the rapid, simultaneous editing of key drivers such as *Trp53*,* Nf1*,* Kras* and *Pten*. These models reproduce key features of human sarcomas, including their histopathology, the initiation of tumours in specific lineages and sites, and tumour–immune interactions within immune-competent hosts. GEMMs have been used to investigate hypotheses about the cells of origin, to test radiotherapy and immunotherapy, and to compare fusion-driven sarcomas with those with a complex karyotype. Despite variability, GEMMs remain essential tools for investigating the mechanisms of initiation, progression, and response to therapy.

**Summary:**

GEMMs offer mechanistic fidelity, but their use is limited by factors such as breeding burden, variability in recombination, off-target effects of CRISPR, underrepresentation of genomic complexity and inconsistent metastasis. These weaknesses reduce their predictive value, particularly with regard to advanced disease and immunotherapy. Progress will require the integration of Cre-loxP with CRISPR-Cas9, the standardisation of induction and reporting, and a closer alignment with distinct sarcoma subtypes, in order to enhance translational relevance.

## Introduction

Sarcomas are divided into two main groups based on their genetic characteristics: simple karyotype sarcomas and complex karyotype sarcomas, each of which presents different challenges in the development of preclinical models. Simple karyotype sarcomas are defined by specific chromosomal translocations that produce unique gene fusions that drive cancer growth [1]. Examples include Ewing’s sarcoma, where an EWS-FLI1 translocation acts as an oncogene; alveolar rhabdomyosarcoma, driven by the PAX3-FOXO1 fusion; and synovial sarcoma, characterised by the SS18-SSX fusion [2]. These sarcomas are easier to model in animals because the disease mechanism is typically linked to a defined genetic alteration [3]. By replicating these fusion proteins in genetically engineered mouse (GEM) models, researchers can closely mimic the human forms of these diseases, allowing reliable testing of treatments and studies of tumour biology [4].

In contrast, sarcomas with complex karyotypes show extensive chromosomal instability with multiple genetic abnormalities, such as amplifications, deletions and rearrangements, without a single driver mutation [5]. This group includes sarcomas such as osteosarcoma, leiomyosarcoma and dedifferentiated liposarcoma. For instance, osteosarcoma, known for its chaotic genome, often has mutations in tumour suppressor genes such as p53 and pRb [6]. To mimic osteosarcoma in preclinical models, researchers often delete these genes in mouse bone cells, resulting in tumours that resemble human osteosarcoma. However, the lack of a single driving mutation means that models can vary in their progression and behaviour, reflecting the diversity of genetic alterations in patients. Dedifferentiated liposarcoma (DDLPS) also often has amplifications of the MDM2 and CDK4 genes [7]. Researchers have used GEM models with MDM2 and CDK4 amplifications to study liposarcoma, but the complex karyotype of DDLPS means that these models don’t capture the full range of mutations seen in patients [8]. However, because complex karyotype sarcomas do not rely on a single genetic alteration, preclinical models for them tend to vary in their progression and behaviour, which may limit the consistency of the models in reflecting human disease. The variability of genetic alterations in complex karyotype sarcomas means that researchers often need to use multiple approaches to develop animal models that can capture the broad genetic diversity seen in patients. One of the studies [9] develops a preclinical model to better understand high-grade complex karyotype sarcomas, which are genetically heterogeneous and associated with poor prognosis. Using a pooled genetic screen based on The Cancer Genome Atlas (TCGA), the researchers identified key genes, in particular YAP1 and wild-type KRAS, as critical drivers of sarcoma development. When these genes were activated in human mesenchymal stem cells (hMSCs), they led to different sarcoma subtypes: YAP1 activation led to undifferentiated pleomorphic sarcoma (UPS), while KRAS activation led to myxofibrosarcoma. In addition, the model demonstrated plasticity by generating leiomyosarcoma and osteosarcoma subtypes when driven by other genes such as CDK4 and PIK3CA. All tumours generated were histologically similar to human sarcomas, with a notable increase in aneuploidy, resembling the complex karyotype of human high-grade sarcomas rather than tumours with a simple karyotype. Gene expression analysis comparing TCGA sarcoma samples with the model tumours revealed increased oxidative phosphorylation signalling in YAP1-driven tumours. The combination of YAP1 inhibition with oxidative phosphorylation inhibitors significantly reduced the viability of several soft tissue sarcoma cell lines, suggesting a promising therapeutic approach.The transcriptional co-analysis of the study shows that YAP1- and KRAS-driven sarcomas may represent a continuum of sarcoma subtypes. This approach not only clarifies subtype relationships within sarcomas, but also identifies new therapeutic targets for these aggressive, genetically diverse cancers. In the development of preclinical models of sarcoma, several key pathways are critical to understanding sarcoma pathogenesis because of their role in tumour growth, genetic stability and treatment resistance. For example, the p53 tumour suppressor pathway is a central regulator in many sarcomas [10, 11]. Although alterations in the *TP53* gene are considered critical drivers of sarcomagenesis, many sarcomas retain wild-type *TP53* but still exhibit phenotypes consistent with loss of p53 function. This observation suggests that disruptions in other elements of the p53 pathway, such as the amplification of Mdm2—a key negative regulator of p53—may lead to functional inactivation of p53 [12, 13]. Both mice and humans with increased Mdm2 levels, caused by a common single nucleotide polymorphism in the Mdm2 promoter (Mdm2SNP309), show a higher risk of developing sarcomas [14–16].Inactivation of p53 has been shown to accelerate tumour formation in genetically engineered mouse models, making it a primary focus of sarcoma research in the Rb pathway: The Rb signalling pathway is frequently altered in sarcomas, particularly osteosarcomas and rhabdomyosarcomas [17]. The Rb protein controls cell cycle progression by inhibiting the transition from G1 to S phase. Loss of Rb function, either through mutations or deletions, removes this control and leads to uncontrolled cell proliferation. In GEM models, Rb loss is often paired with p53 inactivation to create more aggressive and realistic sarcoma models, demonstrating their cooperative role in tumour development [18]. Also, the Ras/MAPK and PI3K/AKT signalling pathways are important for cell survival and proliferation and are often over-activated in sarcomas due to mutations or deletions in genes such as PTEN. In leiomyosarcoma, loss of PTEN leads to activation of the PI3K/AKT pathway, which further destabilises p53 [17]. Mouse models of sarcoma that combine Ras mutations with p53 loss develop tumours rapidly, highlighting the synergy between these pathways in promoting sarcomagenesis [19]. Some of the key pathways in sarcomagenesis along with the examples of studies focused on evaluating this pathway have been summarised in Table 1.

**Table 1:** Key pathways and genes modulated in animal models for sarcomagenesis

Pathway	Key Genes/Translocations	Function/Role in Sarcomagenesis	Animal Models/Disease Types	Example studies using the model	References
*p53 Pathway*	TP53, MDM2, ATM, ATR, p19Arf	Tumour suppressor pathway regulating apoptosis and genomic stability. Loss or mutation of p53 and alterations in MDM2 contribute to sarcoma formation	TP53-mutant mice develop osteosarcoma, rhabdomyosarcoma, and soft tissue sarcomas; MDM2 overexpression used to model liposarcoma​	Walkey et al[20]., Rauch et al[21]., Doyle et al[22]. Smyczynska et al[23].	[10, 15, 24]
*RB Pathway*	RB1, CDKN2A/B, CDK4	Controls cell cycle; deletions/amplifications lead to uncontrolled cell growth, common in sarcomas like osteosarcoma •	RB1-null and CDK4-overexpressing mice model retinoblastoma, osteosarcoma, and leiomyosarcoma​	Dannenberg et al[25].	[26–28]
*Ras/MAPK Pathway*	KRAS, NRAS, HRAS	Promotes proliferation; mutations collaborate with p53 loss in tumour formation, observed in pleomorphic sarcomas​	KRAS-driven models for rhabdomyosarcoma, often combined with TP53 inactivation; HRAS mutations in pleomorphic sarcoma models​	Mito et al[29]., Tsumura et al[29].	[30–32]
*PI3K/AKT/mTOR Pathway*	PIK3CA, PTEN, IGF1R	Pathway activation via mutations/amplifications increases proliferation and invasiveness in sarcoma subtypes​	PTEN-null mice used for rhabdomyosarcoma; PIK3CA and IGF1R overexpression in leiomyosarcoma and liposarcoma models​	Nakamura et al[33]., Cuppens et al. [34].	[35–37]
*WNT/β-Catenin Pathway*	CTNNB1, APC	Regulates differentiation; mutations disrupt normal signalling, leading to tumorigenesis in sarcomas like desmoid tumours​	CTNNB1-mutant models develop desmoid tumours; APC mutations in intestinal adenomas with sarcomatous changes​	Barham et al. [38].	[39–41]
*Chromosomal Translocations*	EWS-FLI1, FUS-DDIT3, PAX3-FOXO1, SS18-SSX1	Fusion proteins disrupt cell cycle regulation, characteristic of sarcomas like Ewing sarcoma and synovial sarcoma​	EWS-FLI1-driven mouse models replicate Ewing sarcoma; PAX3-FOXO1 in alveolar rhabdomyosarcoma; SS18-SSX in synovial sarcoma models​	Haldar et al. [42]., Charytonowicz et al. [43].	[44, 45]
*NF1 Pathway*	NF1, BRCA2	Mutation in NF1 leads to neurofibroma and aggressive tumours like MPNST; BRCA2 mutations implicated in sarcoma susceptibility​	NF1-null mice develop malignant peripheral nerve sheath tumours (MPNST); BRCA2 mutation models in radiation-induced sarcoma​	Dodd et al. [46]., Plante et a [47]l.	[48]
*Chromatin Remodelling*	SMARCB1, EZH2, BCOR	Mutations disrupt chromatin structure, contributing to gene dysregulation in sarcomas like malignant rhabdoid tumours​	SMARCB1-deficient models mimic malignant rhabdoid tumours; BCOR translocation-driven models for undifferentiated sarcoma​	Marques et al. [49].	[50–54]
*Hippo Pathway*	YAP1, TAZ	Dysregulation leads to over-proliferation; implicated in sarcoma development from progenitor cells​	YAP1 and TAZ overexpression models exhibit undifferentiated pleomorphic sarcoma and soft tissue sarcoma​	Ye et al. [55].	[56, 57]
*Apoptosis Pathway*	BAX, BIRC5, SULF1	Regulates programmed cell death; mutations lead to reduced apoptosis, allowing cancer cell survival​	BAX-deficient and BIRC5-overexpressing models in rhabdomyosarcoma and osteosarcoma​	Yan et al. [58].	[59]
*IGF Pathway*	IGF1R, IGF2	Involved in growth and differentiation; upregulated in certain sarcomas, linked to proliferation and survival​	IGF1R and IGF2 overexpression models in rhabdomyosarcoma and liposarcoma​	Lamhamedi-Cherradi et al. [60].	[61]

## Genetically Engineered Mouse

Unlike transplant-based models, GEM models allow researchers to study tumor development from its earliest stages. This helps in understanding how specific genetic changes lead to sarcoma formation and progression. These models are engineered to express or delete specific oncogenes or tumour suppressor genes, enabling researchers to investigate the mechanisms driving tumorigenesis and metastasis [62]. Early GEMs relied on the overexpression of transgenes—either oncogenes or dominant-negative tumour suppressor genes—within specific tissues, utilising ectopic promoter and enhancer elements, such as the immunoglobulin heavy chain enhancer in Eµ–Bcl2 or Eµ–Myc transgenics. The ability to regulate transgene function using exogenous ligands, such as doxycycline for transcriptional control (the Tet system) or tamoxifen for protein function regulation, has enabled temporal control of oncogene expression. This approach has facilitated the demonstration of “oncogene addiction” in specific tissues. For instance, the doxycycline-regulated expression of Kras and Hras has highlighted the roles of these oncoproteins in both the initiation and maintenance of lung cancer and melanoma, respectively. However, these models are still governed by ectopic promoters. GEM models used in sarcoma research previously have been summarised in Table 2.

**Table 2:** Summary of the animal sarcoma models used in preclinical research for the specific subtypes

Sarcoma Subtype	Gene Alteration(s)	Agent Used	Significance	Proposed Karyotype	References
Synovial Sarcoma	SS18-SSX2 fusion combined with SMARCB1 loss	Myf5-Cre	-SMARCB1 loss with SS18-SSX2 expression produces highly aggressive synovial sarcomas. -Loss of SMARCB1 in combination with SS18-SSX2 increases tumour penetrance and affects tumour behaviour. -SMARCB1 reduction disrupts canonical BAF (CBAF) complexes and increases PBAF and GBAF complexes. -The shift to PBAF and GBAF complexes further drives synovial sarcoma development.	Complex, due to the whole-complex degradation of canonical BAF driven by SS18–SSX fusion.	[63, 64]
	Comparison between SS18-SSX1 and SS18-SSX2 fusions	Myf5-Cre, TATCre, and Rosa26-CreER for conditional expression	-Both SS18-SSX1 and SS18-SSX2 independently drive tumour formation, with SS18-SSX2 showing slightly higher sarcomagenic potential and faster onset. -Both fusion genes show high tumour penetrance in synovial sarcoma models. -Exome sequencing indicates few secondary mutations, with SS18-SSX fusions alone being largely sufficient for tumour initiation. -Occasional amplification of chromosome 6 occurs near the fusion gene locus. -Transcriptome analysis shows minimal differences between SS18-SSX1 and SS18-SSX2, suggesting similar functionality.	Translocation with occasional amplification of chromosome 6, highlighting the expression locus for fusion genes.	[65]
	SS18-SSX fusion, with therapeutic targeting of NY-ESO-1	T-cell receptor (TCR) engineered cells (lete-cel) specific for NY-ESO-1 antigen	-Analysed response biomarkers for TCR T-cell therapy targeting NY-ESO-1 in synovial sarcoma. -Higher T-cell doses and fludarabine lymphodepletion correlated with improved therapeutic outcomes. -The therapy significantly remodelled the tumour microenvironment, in particular by reducing macrophage-related gene expression and immunosuppressive factors. -Higher levels of pro-inflammatory cytokines (IL-15, IFNγ) were associated with better responses, while myeloid/macrophage gene expression correlated with resistance.	No additional genetic alterations noted; focus was on immune and gene expression biomarkers within the TME.	[66, 67]
	SS18-SSX2 fusion combined with β-catenin stabilisation	Myf5-Cre and TATCre for inducible gene activation in periosteal cells	-Periosteal cells, bone-adjacent mesenchymal precursors, identified as a potential cell of origin for synovial sarcoma. -SS18-SSX2 fusion, alone or with β-catenin stabilisation, initiates sarcoma formation in periosteal cells. -β-catenin stabilisation accelerates tumour formation and enables sarcoma development in osteoblast precursors. -Osteoprotegerin (OPG) from SS18-SSX2-transformed cells promotes tumour survival close to bone by inhibiting osteoclastogenesis and apoptosis. -Proximity to bone creates a favourable microenvironment that closely associates synovial sarcoma with skeletal sites.	Complex, involving SS18-SSX fusion and β-catenin stabilisation, with no additional chromosomal translocations.	[68]
	SS18-SSX fusion, targeted using NY-ESO-1-specific SPEAR T cells	NY-ESO-1 SPEAR T cells, supported by a lymphodepletion regimen with fludarabine and cyclophosphamide	-Improved outcomes with high NY-ESO-1 expression and fludarabine-based conditioning. -IL-7 and IL-15 levels after conditioning supported T cell persistence. -Consistent NY-ESO-1 expression after therapy with minimal loss. -High levels of CD163 + macrophages maintained an immunosuppressive environment.	Complex, with no additional structural changes reported; focuses on TME interactions and immune factors.	[69]
Malignant Peripheral Nerve Sheath Tumours (MPNSTs)	*Nf1 deletion combined with Ink4a/Arf deletion*,* and Nf1 deletion with p53 deletion*	No additional induction agents; relies on genetic mutations	-The frequency of tumour-initiating cells is genotype dependent, with Nf1+/-; Ink4a/Arf-/- mice having a higher proportion of tumour-initiating cells than Nf1+/-; p53+/- mice. -Nf1+/-; p53+/- MPNST cells require exogenous laminin for growth, whereas Nf1+/-; Ink4a/Arf-/- cells show endogenous laminin expression. -Laminin binding to β1-integrin promotes survival and proliferation, whereas laminin expression enhances clonogenic potential. -Genotype-specific responses to assay conditions affect the frequency of detection of tumourigenic cells.	Complex, influenced by genetic and environmental factors.	[43]
Soft Tissue Sarcoma (STS) and Malignant Peripheral Nerve Sheath Tumour (MPNST)	Kras activation (G12D mutation) and Trp53 knockout in STS; Nf1 and Trp53 knockout in MPNST	Adenovirus expressing Cre recombinase and sgRNA targeting Trp53 for intramuscular injection into KrasLSL-G12D/+; Rosa26LSL-Cas9-EGFP/+ mice; Cas9 and sgRNA targeting Nf1 and Trp53 for sciatic nerve injection into wild-type mice.	-CRISPR/Cas9 combined with Cre-lox recombination enabled rapid generation of primary sarcomas with tumour development kinetics similar to GEMMs using the Cre-loxP system alone. -This approach bypassed the need for extensive breeding in GEMMs, allowing faster and less costly sarcoma modelling in vivo. -Demonstrates the utility of CRISPR/Cas9 for preclinical sarcoma research, enabling rapid assessment of tumour-modifying genes in both GEMMs and wild-type mice.	Complex, due to targeted *Kras* mutation and *Trp53*/*Nf1* deletions.	[70]
	Wnt1-Cre, 3.9Periostin-Cre, P0a-Cre	Nf1 deletion alone or in combination with Ink4a/Arf and p53 deletions	-While Nf1 deletion increases the self-renewal capacity of neural crest stem cells (NCSCs), it alone does not lead to tumour formation. However, additional p53 or Ink4a/Arf mutations lead to MPNSTs in adult mice. -NCSCs show only transient self-renewal upon Nf1 loss and do not persist postnatally; tumours are likely to arise from differentiated glial cells. -Specific Cre drivers allow Nf1 deletion in Schwann cell lineages, leading to abnormal Schwann cell proliferation and eventual tumour formation in adulthood.	Complex, involving deletions and mutations in *Nf1*, *p53*, and *Ink4a/Arf* pathways.	[71]
Uterine Leiomyosarcoma	Overexpression of Cripto-1 (CR-1)	MMTV-LTR promoter	−19.7% of MMTV-CR-1 female mice developed uterine leiomyosarcomas, while none occurred in the control group, indicating a significant tumourigenic effect of CR-1. -Tumours showed increased levels of phosphorylated (P) src, Akt, GSK-3β and nuclear localisation of β-catenin, indicating activation of CR-1 and canonical Wnt signalling pathways. -Human uterine leiomyosarcomas also showed high CR-1 expression and similar pathway activation, suggesting that CR-1 and Wnt signalling are critical in tumorigenesis.	Complex, with focus on pathway activation rather than additional chromosomal alterations.	[72]
Osteosarcoma	p53R172H mutation, equivalent to the human p53R175H hot spot mutation	Cre-loxP system for targeted p53 mutation (knock-in model)	-The p53R172H mutation leads to an increased incidence of osteosarcoma with a significant increase in metastatic spread to organs such as the liver, lung and brain. -In contrast to p53 loss of function, the p53R172H mutation drives cell proliferation and transformation through a gain of function effect. -The mutant p53 protein binds and inactivates p53 family members p63 and p73, promoting cell survival and transformation, which is likely to contribute to the tumourigenic phenotype.	Complex, due to the combined effects of the mutant p53 on cellular pathways and interactions with p53 family members.	[73]
	Trp53 and Rb deletions in osteoblast progenitors	Osx-Cre for conditional Trp53 and Rb deletion in osteoblasts	-Combined Trp53 and Rb deletions resulted in rapid and highly penetrant osteosarcoma formation, with mice displaying early-onset malignant osteosarcoma. -This model recapitulates key features of human osteosarcoma, including cytogenetic complexity, similar gene expression profiles and metastatic potential, making it highly relevant for the study of osteosarcoma. -Tumours frequently arose in the jaw, ribs and hind limbs, with pathology similar to human osteosarcoma, including metastasis, particularly to the lungs.	Complex, with structural abnormalities similar to human osteosarcoma.	[20]
	Prkar1a deletion, inducing deregulated PKA signaling	Transgenic SV40 T antigen under the control of the osteocalcin promoter to induce Prkar1a deletion in bone tissue	-Loss of Prkar1a resulted in a subclass of osteosarcoma with increased RANKL expression, promoting an osteoclastogenic microenvironment and increased metastatic potential, primarily to the lung. -Prkar1a functions as a bone-specific tumour suppressor and its deletion accelerates osteosarcoma development in mouse models, producing aggressive, high-grade osteoblastic lesions. -The absence of Prkar1a deregulates PKA signalling, identified as a critical pathway in osteosarcoma pathogenesis, further affecting the RANKL/OPG ratio and facilitating tumour proliferation.	Genomic instability inducing high levels of phospho-CREB and RANKL expression with complex karyotypes and recurrent Prkar1a deletion.	[74]
	Trp53 and Rb1 deletions	Cre-loxP system with different Cre drivers (Prx1-Cre, Col1α1-Cre, Oc-Cre) to target distinct osteoblast differentiation stages	-Tumours arose from multiple osteoblastic lineages, including differentiated, minimally proliferative osteoblasts, suggesting that even late-stage osteoblasts can undergo transformation. -Mineralisation and osteoid matrix production did not correlate with the differentiation state of the cell of origin, suggesting extensive dedifferentiation during tumour formation. -Silencing of DNA methyltransferases correlated with dedifferentiation, supporting the role of epigenetic reprogramming in osteosarcomagenesis.	Complex, due to *Trp53* and *Rb1* inactivation across various stages of osteoblast differentiation.	[75]
Osteosarcoma, with high metastatic potential	Rb and p53 inactivation	Osx1-Cre transgene targeting osteoblast precursors	-Combined Rb and p53 inactivation resulted in the development of osteosarcoma in 75% of mice, with a significant rate of metastasis to organs such as the lung and liver. -The tumours exhibited typical features of human osteosarcoma, including poor differentiation, the presence of osteoblastic cells and the potential for both bone and adipocyte differentiation. -Tumorigenicity correlated with the expression of Sca-1, a marker associated with stem cells and progenitor cells, suggesting a role for mesenchymal progenitor cells in tumour initiation.	Complex, involving dual inactivation of *Rb* and *p53* leading to aggressive and metastatic tumour behaviour.	[76]
Osteosarcoma, specifically osteoblastic and fibroblastic subtypes	Trp53 deletion, Rb1 deletion, and p53 knockdown via shRNA	Osx-Cre for osteoblast-specific Cre deletion; tetracycline-regulated shRNA targeting p53	-Trp53 and Rb1 deletions resulted in fibroblastic osteosarcomas, whereas shRNA-mediated p53 knockdown resulted in osteoblastic osteosarcomas, reflecting distinct human subtypes. -The shRNA-driven osteoblastic model showed higher lung metastasis rates, similar to metastatic patterns in human osteosarcoma. -Comparison of Cre and shRNA knockdown methods showed different tumour phenotypes, highlighting the different outcomes based on the genetic approach.	Complex, involving deletions and transcriptional knockdown affecting differentiation and tumour phenotype.	[77]
Osteosarcoma, with subtypes including osteoblastic, fibroblastic, and chondroblastic	Apc1638N mutation and Twist haploinsufficiency	Genetic mutation model (Apc; Twist double heterozygotes)	-Mice with Apc and Twist alterations spontaneously developed osteosarcomas in various skeletal regions, with tumours displaying histological subtypes commonly seen in human osteosarcomas. -Osteolytic lesions with active osteoblastic markers were identified, mimicking human osteosarcomas, particularly craniofacial osteosarcomas. -The model showed downregulation of Runx2 and variable pRb expression across tumour subtypes, consistent with deregulation observed in human osteosarcoma.	Complex, with osteosarcoma development driven by concurrent *Apc* loss and *Twist* haploinsufficiency.	[78]
Osteosarcoma, specifically the osteoblastic subtype	Ptch1 deletion with p53 heterozygous mutation	HOC-Cre for osteoblast-specific Ptch1 deletion and partial upregulation of Hedgehog signaling in mature osteoblasts	-Activation of Hh signalling in osteoblasts led to overexpression of Yap1 and H19, critical oncogenes in osteosarcoma pathogenesis, and mimicked human osteoblastic osteosarcoma characteristics. -Ptch1 deletion with p53 heterozygosity resulted in early-onset osteosarcoma with frequent metastasis, particularly to the lungs, and demonstrated aggressive tumour behaviour. -The findings highlight Hh signalling as a key driver of osteosarcoma, with potential therapeutic implications in targeting Yap1 and H19 expression.	Complex, involving Hedgehog pathway dysregulation combined with *p53* tumor suppression compromise.	[79]
Osteosarcoma/hemangiosarcoma	p53R270H and p53R172H mutations (analogous to human p53R273H and p53R175H hot spot mutations)	Conditional Cre-LoxP system to induce specific point mutations in p53	-Mice with p53R270H and p53R172H mutations developed a range of sarcomas and carcinomas, with the p53R172H mutation leading to more frequent osteosarcomas and metastases. -Both mutations showed gain-of-function effects, promoting different tumour types beyond those observed in p53-null mice. -Mutant p53 proteins showed dominant-negative effects on wild-type p53, with additional oncogenic properties driving tumorigenesis.	Complex, due to diverse oncogenic roles of mutant *p53* in tumour formation and progression.	[80]
Ewing’s Sarcoma and other poorly differentiated sarcomas	EWS-FLI1 fusion, conditional p53 deletion	Prx1-Cre for limb mesenchyme-specific EWS-FLI1 expression and p53 deletion	-Expression of EWS-FLI1 in limb mesenchyme caused severe limb shortening and musculoskeletal abnormalities. -EWS-FLI1 in combination with p53 deletion led to rapid onset of poorly differentiated sarcomas, reducing tumour latency from 50 to 21 weeks. -p53 deletion alone resulted primarily in osteosarcomas, whereas EWS-FLI1 expression shifted the tumour type to poorly differentiated sarcomas resembling human Ewing’s sarcoma.	Complex, driven by fusion gene expression and loss of tumor suppression pathways.	[81]
Ewing Sarcoma	EWS-FLI1 fusion gene	CNI-1493 (semapimod), a macrophage inhibitory compound	-CNI-1493 treatment significantly reduced metastatic tumour burden in a mouse model of Ewing sarcoma by inhibiting M2 macrophage-stimulated tumour cell invasion and extravasation. -The drug specifically targets M2 polarised macrophages associated with tumour invasion and metastasis. Inhibition of extravasation: In vivo, CNI-1493 reduced the presence of metastatic foci in the lung by preventing tumour cells from invading the lung parenchyma. -The study suggests that CNI-1493 may serve as a therapeutic agent to control metastatic spread in Ewing sarcoma, particularly by limiting TAM-driven metastasis.	Focused on EWS-FLI1 fusion as the primary oncogenic driver.	[82]
	EWS-FLI1 fusion gene expression using various tissue-specific promoters	Transgenic expression under promoters like Runx2, Osterix, Prx1, and Col1a1 targeting osteoblast precursors and mesenchymal tissues	-Attempts to express EWS-FLI1 in various tissues have resulted in embryonic lethality, developmental defects or alternative tumours such as fibrosarcoma instead of ES. -The study found that successful ES induction may require permissive cells and specific developmental timing, as broad EWS-FLI1 expression often triggered apoptosis or failed to induce tumourigenesis. -Highlights the difficulty of achieving EWS-FLI1 expression without off-target effects or cytotoxicity, suggesting the need for refined, lineage-specific targeting strategies in ES modelling.	Complex driven by EWS-FLI1 fusion with variable cell type and promoter specific effects.	[83]
Clear Cell Sarcoma (CCS)	EWSR1::ATF1 fusion gene	Inducible V5-tagged EWSR1::ATF1 expression in mice, driven by the Rosa26 promoter with Cre-mediated activation.	-The mouse CCS tumours closely mirrored the human CCS transcriptome, including shared gene expression signatures and differentially expressed genes involved in proliferation and metabolic reprogramming. -EWSR1::ATF1 bound to super-enhancers (SEs) that regulate critical oncogenes, highlighting its role in epigenetic reprogramming. -EWSR1::ATF1 preferentially bound promoter regions with canonical ATF/CREB motifs and distal enhancer regions with novel TGA repeat motifs, similar to the enhancer binding activity of EWSR1::FLI1 in Ewing sarcoma. -The model validated SEs as core regulatory chromatin features of CCS and identified genes such as CREM and NFIL3 as potential oncogenic drivers.	Complex, driven by EWSR1::ATF1 fusion-mediated transcriptional and epigenetic dysregulation.	[84]
Peripheral Chondrosarcoma (PCS)	Mosaic Ext1 loss with additional deletion of Trp53 or Ink4a/Arf	Col2-rtTA-Cre system to induce conditional Ext1, Trp53, or Ink4a/Arf deletions in chondrocytes	-Mice with mosaic Ext1 loss combined with Trp53 or Ink4a/Arf deletion developed aggressive chondrosarcoma-like lesions, mimicking human PCS. -PCS tumours showed disrupted chondrocyte polarity and ciliogenesis, resembling the invasive growth pattern of human PCS. -Both Trp53 and Ink4a/Arf deletions were sufficient to drive progression from osteochondroma to malignant chondrosarcoma.	Complex, with gene inactivation leading to dysregulated chondrocyte growth and polarity.	[85]
Mesenchymal Chondrosarcoma (MCS)	HEY1-NCOA2 fusion gene	Retroviral transduction of HEY1-NCOA2 in embryonic superficial zone (eSZ) chondroprogenitor cells, followed by subcutaneous transplantation into nude mice	-HEY1-NCOA2 expression in eSZ cells induced tumours with biphasic morphology typical of human mesenchymal chondrosarcoma, including Sox9 and Runx2 expression associated with chondrogenesis. -Runx2 deletion delayed tumour onset and resulted in a predominance of immature small round cells, demonstrating that Runx2 supports differentiated cartilage morphology in MCS. -Panobinostat (HDAC inhibitor) effectively suppressed tumour growth in vitro and in vivo, suggesting potential therapeutic efficacy for targeting HDAC-regulated pathways in HEY1-NCOA2-driven MCS.	Complex driven by HEY1-NCOA2 fusion that affects chondrogenic differentiation pathways.	[86]
Alveolar Rhabdomyosarcoma (ARMS)	Pax3 fusion combined with p53 or Cdkn2a deletions	Myf6-Cre for gene recombination in maturing myofibers	-The Pax3 fusion with biallelic p53 or Cdkn2a inactivation resulted in ARMS with 100% penetrance and rapid progression, mimicking human ARMS. -Tumours frequently exhibited distant metastasis, particularly in p53 inactivation models, resembling the aggressive metastatic nature of human ARMS. -Gene expression analyses confirmed that the mouse ARMS model shares a conserved molecular signature with human ARMS, supporting its utility in the study of disease mechanisms and therapeutic targets.	Complex, involving fusion and inactivation of key tumour suppressor genes.	[87]
Rhabdomyosarcoma (RMS), including alveolar (fusion-positive) and embryonal (fusion-negative) subtypes.	Elevated INSR-A isoform expression due to alternative splicing of the Insr pre-mRNA.	Antisense oligonucleotides (SSOs) targeting Insr splicing to shift from INSR-A to INSR-B isoform. Delivery in RMS cell lines and a CRISPR-generated Insr-splicing mouse model.	-SSO treatment reduced RMS cell proliferation and migration in vitro. -Targeting INSR-A reduced pAKT signalling and angiogenesis. -In vivo, SSOs modified Insr splicing in muscle tissue, supporting therapeutic potential for RMS.	Driven by *Insr* alternative splicing with elevated INSR-A isoform promoting tumorigenesis in RMS.	[88]
Kaposi Sarcoma	Full KSHV genome integration	Bacterial Artificial Chromosome (BAC) with the entire KSHV genome injected into mouse oocytes	-Mice developed an aggressive, early-onset angiosarcoma that was histologically and molecularly similar to human Kaposi sarcoma. -Tumours expressed latency-associated nuclear antigen (LANA) and other KSHV viral genes, activating pathways (PI3K/Akt/mTOR, VEGF, IL-10) similar to human KS. -Stable transmission of the KSHV transgene resulted in reproducible tumour formation and provided a model for KS without additional mutations or co-infections.	Complex, driven by viral oncogene expression and pathway activation rather than chromosomal rearrangements.	[89]
Gastrointestinal Stromal Tumours (GIST)	KitV558Δ mutation in exon 11, constitutive activation	Site-directed mutagenesis introducing a *Kit* exon 11 mutation; EIIa-Cre transgenic mice for *neo* cassette excision.	-The KitV558Δ mutation leads to constitutive activation of the Kit receptor, resulting in hyperplasia of Kit-positive interstitial cells of Cajal (ICC) and spontaneous GIST formation. -GISTs develop in almost all KitV558Δ heterozygous mice, particularly in the cecum, closely resembling human familial GISTs. -Mutant mice show increased mast cell numbers in the skin and GI pathology, mirroring human GIST-associated conditions.	Complex, focused on a single mutation leading to oncogenesis.	[90]
	Kit K641E mutation	Knock-in mutation of *Kit* gene via site-directed mutagenesis	-Both homozygous and heterozygous Kit K641E mice developed GISTs, with the mutation leading to complete penetrance in homozygotes, resulting in gastrointestinal obstruction and early death. -Extensive hyperplasia of interstitial cells of Cajal (ICC), particularly affecting the pylorus and cecum, closely models the human familial GIST syndrome. -The Kit K641E mutation causes constitutive phosphorylation of Kit, which supports ICC proliferation and tumour growth. -Homozygotes exhibited white coat colour, decreased dermal mast cells and sterility, indicating partial loss of normal Kit function despite oncogenic activation.	Complex, due to the singular activating mutation impacting both oncogenic and functional pathways.	[91]
Undifferentiated Pleomorphic Sarcoma (UPS)	Trp53 and Pten homozygous deletions	Adeno-Cre recombinase injection for targeted gene recombination	-Injection of Ad-Cre into Trp53fl/flPtenfl/fl mice resulted in UPS formation with 100% penetrance, primarily at the injection site. -The model incorporates a luciferase reporter for real-time bioluminescence imaging (BLI), allowing non-invasive monitoring of tumour development. -Tumours showed lymphocyte infiltration in 64% of cases and upregulated PD-L1 in 71%, suggesting an immunosuppressive microenvironment. -Although UPS was the predominant type, a minority of tumours differentiated into pleomorphic rhabdomyosarcoma (PRMS).	Complex, involving *Trp53* and *Pten* inactivation and associated immunogenic modulation.	[92]
	Kras activation (G12D) and p53 deletion in KP model; p53 deletion with MCA mutagen in high-mutational load model	Adeno-Cre injection to induce *Kras* and *p53* alterations in LSL-*Kras*G12D;*p53*Flox/Flox (KP) mice; additional MCA treatment for high-mutational load *p53*Flox/Flox model	Metastasis rates: In the KP model, approximately 40% of the mice developed lung metastases after amputation, while only ~ 12% metastasised in the p53/MCA model. Metastasis regulators: Genetic analyses identified miR-182, NEAT-1 and HIF-1a as key regulators of lung metastasis in sarcoma. Impact on the immune system: Despite low metastasis in the highly mutated model, low metastasis rates persisted even in Rag2-/- mice, suggesting that MCA mutations may disable metastasis-promoting intrinsic factors. -Lineage tracing and CRISPR-generated barcoding revealed that specific gene expression profiles in clones correlated with lung metastatic potential.	Complex, with Kras-driven tumorigenesis in KP model and high mutational burden from MCA in *p53*/MCA model.	[62]
	KrasG12D activation and Trp53 deletion (KP model)	Adenovirus expressing CMV-Cre injected into the gastrocnemius muscle of *Rosa26*tdTomato; *KrasG12D/+; p53Flox/Flox* mice. tdTomato + lineage tracing for identifying tumour cells.	UPS tumours exhibited significant intratumoral heterogeneity in histology and transcriptional state. Even within the same mouse, independent tumours had different dominant cell populations, covering a wide phenotypic space. Immunohistochemical staining confirmed the variability in the expression of markers such as Col1a1 (collagen type I alpha 1 chain) and desmin. UPS tumours were much more heterogeneous than lung adenocarcinomas derived from the same GEMM, highlighting the sarcoma-specific diversity. Differential expression of imprinted genes such as Meg3 (maternal) and Peg3 (paternal) suggested differential transcriptional regulation within tumour subpopulations. Key pathways such as epithelial-mesenchymal transition (EMT) and myogenesis were enriched in different subgroups. The analysis revealed three major transcriptional subpopulations within tumours, likely reflecting different differentiation states. The subpopulations may explain phenotypic and functional differences observed in tumour histology.	Complex, driven by *KrasG12D* activation and *p53* loss, with additional transcriptional and epigenetic alterations contributing to heterogeneity.	[93]
	KrasG12D activation, Pten and Trp53 deletion	Spontaneous tumour model: KrasG12D activation, Pten deletion and Trp53 deletion were introduced by Cre-mediated recombination into genetically engineered mice. Cre recombinase was delivered using an adenovirus injected intramuscularly into targeted tissues to induce specific genetic changes. Transplanted tumour model: Tumours were derived from cell lines with KrasG12D/Pten/Trp53 (KPP) or KrasG12D/Trp53 (KP) alterations and implanted into immunocompetent syngeneic hosts.	Spontaneous tumours: Showed higher immune infiltration and inflammatory signatures compared to transplanted tumours. Engrafted tumours: Better replicated the immune cold phenotype typical of UPS. Immune microenvironment: Spontaneous tumours in the KrasG12D/Pten/Trp53 (KPP) model showed increased lymphocyte activation and immune infiltration compared to the KrasG12D/Trp53 (KP) model. Molecular pathways: Differentially enriched gene sets associated with immune response, stem cell pluripotency and tumour growth pathways.	Complex, driven by *KrasG12D* mutation and *Trp53/Pten* deletions with extensive immune and inflammatory signalling differences.	[94]
Pleomorphic Liposarcoma	Trp53, Rb1, and Pten deletions	TAT-Cre injection in limb muscle to induce gene silencing	-Mice developed pleomorphic Liposarcomas within 6 weeks of injection, with rapid tumour progression between 100 and 150 days. -Tumours closely resembled human pleomorphic liposarcoma, characterised by pleomorphic lipoblasts. -Tumours exhibited an immune profile with prominent CD8 + T cell and macrophage infiltration, similar to the immune landscape of soft tissue sarcomas. -Given the intact immune response and tumour similarity to human counterparts, this model could be valuable for testing immunomodulatory treatments.	Complex, resulting from loss of *Trp53*, *Rb1*, and *Pten* tumour suppressors.	[95]
Dedifferentiated Liposarcoma (DDLPS)	Trp53 and Pten deletions	Adipocyte-specific *Cre*-mediated deletion of *Trp53* and *Pten* via AAV8-Cre in C57BL/6 mice (ACPP model). Immune checkpoint blockade (ICB) with anti-PD1 and anti-LAG3 therapies.	-Histological and transcriptomic analyses confirmed that the ACPP model recapitulated human DDLPS. -Similar hallmark pathways were observed in mouse and human tumours, including dysregulated P53 and AKT signalling. -Adipocyte-specific *Cre*-mediated deletion of *Trp53* and *Pten* via AAV8-Cre in C57BL/6 mice (ACPP model). -Immune checkpoint blockade (ICB) with anti-PD1 and anti-LAG3 therapies. -Tumours were categorised into DDLPS_low (< 20% CD3 + T cells) and DDLPS_high (> 20% CD3 + T cells) groups. -DDLPS_high tumours had significantly higher levels of CD8 + T cells, FOXP3 + regulatory T cells and PD1 + exhausted T cells. -Among the syngeneic cell-derived tumours (N1011, N1018, N1343): -N1018 tumours showed delayed growth with ICB, which was associated with a 3-fold increase in CD8 + T cells (p adj. < 0.01). -Pre-treatment CD69+/CD103 + resident memory T cells (TRM) associated with ICB response. -N1011 and N1343 tumours did not respond to anti-PD1 or anti-LAG3.	Complex driven by chromosome 12q13-15 amplification, with MDM2 overexpression and Trp53/Pten deletions contributing to tumourigenesis.	[96]
Myxoid Liposarcoma	FUS-CHOP fusion	EF-1a promoter to drive FUS expression; combined with CHOP transgene in double-transgenic FUSxCHOP mice	-FUS-CHOP fusion results from a t(12;16) translocation, creating a fusion gene critical for liposarcoma formation; FUS domain contributes to oncogenesis by acting as a transcriptional activator. -Double transgenic FUSxCHOP mice developed liposarcomas with cellular features similar to human liposarcomas, confirming the role of FUS-CHOP fusion in liposarcoma pathogenesis. -FUS-CHOP expression blocks adipocyte differentiation and contributes to tumour formation by inhibiting differentiation.	Complex, with specific chromosomal translocation and fusion gene formation.	[97]
Leiomyosarcoma (LMS)	Loss of Usp18 gene.	Genetically engineered Usp18-/- mice developed LMS spontaneously; tumors were transplanted subcutaneously into syngeneic FVB/N mice.	−25.6% of Usp18-/- mice developed palpable LMS tumours within ~ 210 days. -Morphology and immunohistochemistry (IHC) confirmed the LMS histotype, consistent with human LMS. -RNA sequencing revealed transcriptional profiles in one third of mouse tumours that closely matched human LMS datasets, including TCGA.	Complex, driven by Usp18 loss, with conserved LMS-related gene signatures in human and mouse samples.	[98]
Angiosarcoma	Trp53 deletion and Trp53R172H mutation	Cdh5-CreERT2 for endothelial-specific p53 loss; Pdgfrb-Cre for pericyte-specific p53 targeting	-Complete Trp53 deletion in endothelial cells using Cdh5-CreERT2 resulted in angiosarcoma development in all mice with a median Lifespan of 325 days. -Mice with Trp53R172H mutations predominantly developed thymic lymphomas; however, the Trp53R172H mutation under Pdgfrb-Cre drove angiosarcoma formation. -Cdh5-CreERT2 models produced angiosarcomas specifically in endothelial tissues, whereas Pdgfrb-Cre models occasionally produced undifferentiated sarcomas, highlighting the different oncogenic potentials of p53 loss versus mutation. -Angiosarcoma tumour fragments from Cdh5-CreERT2, Trp53fl/fl mice were successfully transplanted in vivo, providing a consistent model for drug testing.	Complex, with distinct tissue-specific outcomes for *p53* deletion versus *p53* mutation.	[99]
	Trp53 loss (homozygous deletion) or Trp53 R172H mutation	Cdh5-Cre for endothelial-specific Trp53 deletion or mutation; Pdgfrb-Cre targeting pericytes and endothelial cells.	-In Cdh5-Cre, Trp53fl/fl mice, 100% developed angiosarcomas with a median Lifespan of 325 days. In Pdgfrb-Cre, Trp53R172H/R172H mice, 75% developed angiosarcomas, while others developed lymphomas or teratomas. -Angiosarcomas showed typical pleomorphic features, vascular formations and expressed CD31 and ERG, markers typical of endothelial tumours. -Tumour fragments were successfully transplanted from Cdh5-Cre, Trp53fl/fl mice, providing a model suitable for serial transplantation for therapeutic testing. -The Trp53 R172H gain-of-function mutation resulted in a distinct gene expression profile, indicating different oncogenic pathways compared to Trp53 deletion.	Complex, reflecting *Trp53* mutation/deletion impacting endothelial and pericyte lineages.	[100]
	Tp53 knockout via T273X mutation in DNA-binding domain.	Tp53-deficient Wistar rats developed sarcomas spontaneously or upon heterozygous Tp53 loss.	-Homozygous knockout rats developed predominantly angiosarcomas with pulmonary metastases at four months of age. -Transcriptomic profiling identified over 3000 differentially expressed genes, highlighting upregulated cell cycle pathways (mitosis, DNA replication) and downregulated metabolic pathways (TCA cycle, fatty acid metabolism). -Tumours lacked major chromosomal rearrangements, with limited aneuploidy compared to heterozygous mutants.	Driven by Tp53 loss with minimal secondary chromosomal changes, highlighting differences in sarcomagenesis mechanisms between homozygous and heterozygous mutations.	[23]
Soft Tissue Sarcoma (STS)	KrasG12D activation and Trp53 deletion (KP model)	X-PACT Therapy: Combination of 8-methoxypsoralen (8-MOP) and phosphors injected intratumorally. Activation Protocol: Low-dose X-ray radiation to locally activate psoralen through phosphor-generated UV light.	-Treatment significantly increased tumour necrosis. -Preliminary micro-CT imaging showed a measurable reduction in tumour size over the treatment period. -G-CSF, eotaxin, TIMP-1 and IL-1β levels were significantly reduced in treated mice, suggesting a potential suppression of systemic tumour-promoting inflammation. -Increased MDC suggests localised immune activation, potentially favouring an antitumour response. -Hematoxylin and eosin (H&E) staining revealed increased necrotic areas in treated tumours compared to controls. -X-PACT showed early promise for local tumour control and immune modulation. -The abscopal effects observed in clinical trials in dogs suggest a potential systemic effect on metastasis and warrant further investigation in mouse models.	Complex, with oncogenic Kras activation and *Trp53* loss driving tumour initiation.	[101]
Sarcoma, primarily osteosarcoma (OS) and poorly differentiated soft tissue sarcoma (PDSTS)	p53 homozygous deletion, Rb homozygous deletion, and combined p53/Rb deletions	Prx1-Cre and Col1a1-Cre for targeted gene deletion in mesenchymal cells and osteoblast lineage	-Prx1-Cre-mediated p53 deletion resulted in high sarcoma penetrance, with osteosarcoma (61%) and PDSTS (32%) as primary tumour types. -p53 deletion alone induced sarcomas, whereas Rb deletion alone did not; combined p53 and Rb deletions accelerated sarcoma onset and increased PDSTS prevalence. -Prx1-Cre targeted early mesenchymal cells, suggesting these undifferentiated cells as potential sarcoma precursors, whereas Col1a1-Cre targeted p53 deletion in committed osteoblasts, resulting predominantly in osteosarcomas.	Complex, with significant roles for *p53* loss and *Rb* loss in mesenchymal cell tumorigenesis.	[99]
Various sarcomas, including hemangiosarcoma, osteosarcoma, and rhabdomyosarcoma	Mdm2 overexpression	Mdm2-transgenic model using the entire Mdm2 gene with its native promoter	-High Mdm2 levels induced spontaneous tumour formation independent of p53, specifically increasing sarcoma incidence. -Transgenic mice developed a higher incidence of sarcomas, including hemangiosarcoma and osteosarcoma, compared to non-transgenic controls. -Sarcoma incidence and tumour onset accelerated with increased Mdm2 copy number, indicating a dose-response relationship.	Complex, focused on gene amplification rather than additional chromosomal alterations.	[102]

### Recombination Methods

**Fig. 1:**
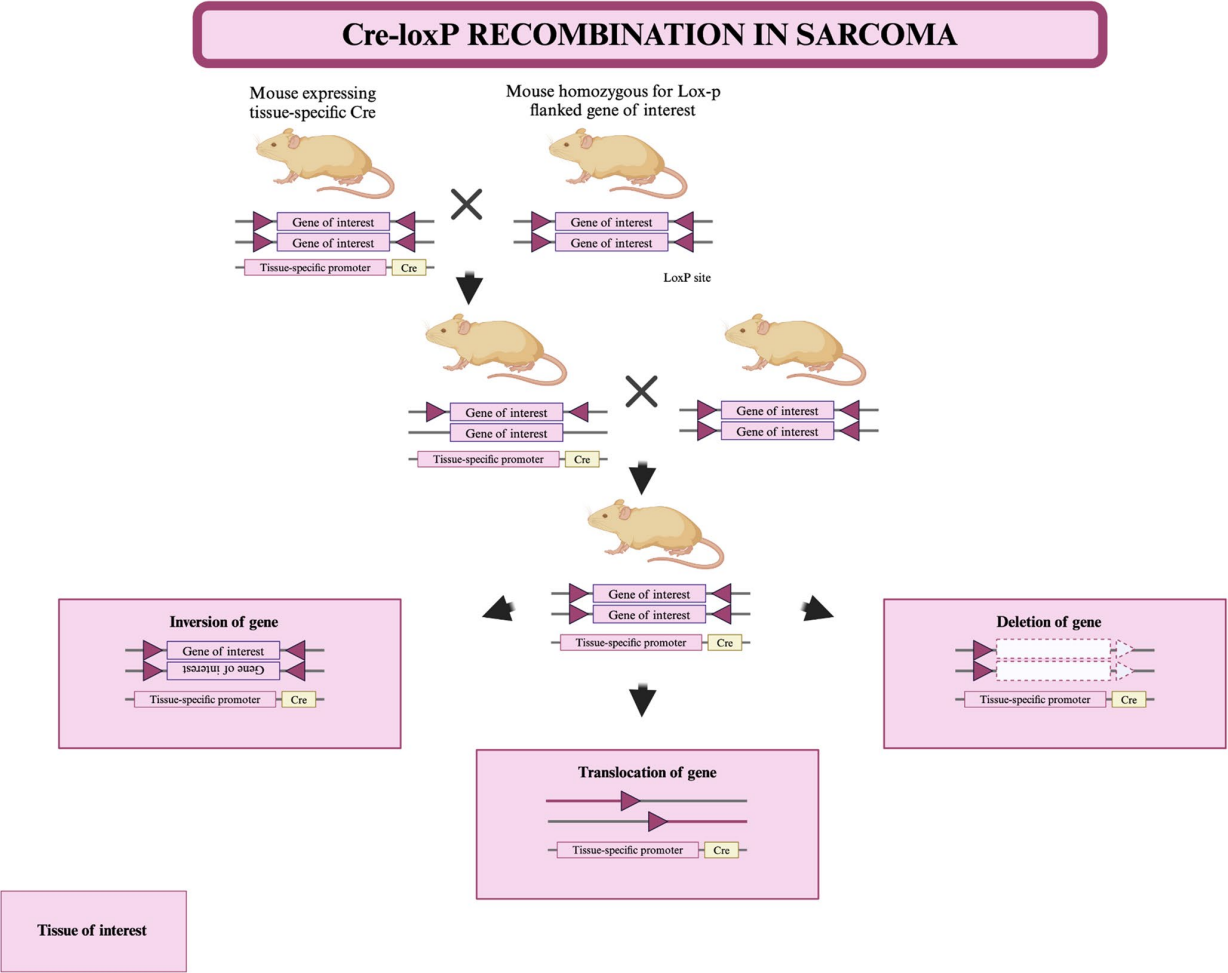
Cre-loxP recombination mechanism in sarcoma research

There are several methods for creating GEMMs to study sarcomas and other cancers. Two of the most widely used approaches are Cre-loxP recombination and CRISPR-Cas9 genome editing [103]. Each method offers unique advantages and has specific mechanisms that make them suitable for different experimental designs. Cre-loxP recombination uses the Cre recombinase enzyme to recognise specific loxP sites in DNA. This system allows site-specific recombination, enabling researchers to knock out or activate genes in a controlled manner. By placing loxP sites around a target gene and introducing Cre recombinase via tissue-specific or inducible promoters, mutations can be restricted to specific tissues or developmental stages [104] (Fig. 1). This method is widely used to model genetic conditions that require precise spatial or temporal control of mutations. For instance, in a study [105]aiming to evaluate the relative biological effectiveness (RBE) of carbon ion therapy (CIT) compared to X-ray therapy in treating soft tissue sarcomas. The GEM model, based on LSL-KrasG12D; p53fl/fl (KP) mice, was specifically designed to replicate the genetic and biological complexity of human undifferentiated pleomorphic sarcomas. Cre recombinase was delivered by adenoviral injection directly into the gastrocnemius muscle, allowing precise genetic modification. This process excised the transcriptional stop cassette upstream of the mutant KrasG12D allele, activating its expression, while simultaneously deleting both alleles of p53, a critical tumour Suppressor gene. This approach induced localised tumourigenesis in a physiologically relevant environment, allowing the tumour to develop within its native tissue microenvironment and co-evolve with the host immune system. CIT was delivered using a monoenergetic beam shaped to produce a 3 cm spread-out Bragg peak (SOBP), while X-ray therapy was delivered using precise image-guided radiation techniques. The study compared Single doses of 20 Gy, 25 Gy and 30 Gy of X-rays with a Single 10 Gy dose of CIT. Tumour volume, proliferation markers (e.g. Ki-67), mitotic activity (phospho-histone H3) and immune infiltration (CD3 + T cells) were analysed to assess treatment response and tumour kinetics. The results showed that a 10 Gy dose of carbon ions was as effective as 30 Gy of x-rays in delaying tumour growth, leading to an RBE calculation of 3. CIT also induced a significantly higher T-cell infiltrate compared to isoeffective x-ray doses, highlighting enhanced immune activation. Tumours treated with CIT exhibited greater mitotic activity at the endpoint compared to those treated with X-rays, suggesting different patterns of recurrence. Notably, CIT-treated tumours experienced a longer period of growth arrest, but showed faster regrowth upon recurrence compared to X-ray-treated tumours. The GEM model was chosen over syngeneic or xenograft models because of its superior ability to mimic the natural co-evolution of the tumour and its host immune system.

However, Cre-loxP has its limitations for example use depends on the prior incorporation of loxP sites flanking the target gene or genomic region, which requires extensive genetic engineering [106]. This process can be labor-intensive, especially for generating transgenic animals, and may involve breeding multiple lines to achieve the desired recombinase activity. Once loxP sites are in place, the recombination efficiency can vary based on the genomic context, such as chromatin accessibility, proximity to transcriptionally active regions, or the sequence composition near the loxP sites. Inefficient recombination may lead to incomplete or mosaic deletions, particularly in tissues with low Cre expression or poor Cre delivery. Off-target recombination can also be challenging as it can occur if cryptic loxP-like sequences are present elsewhere in the genome, resulting in unintended gene disruptions [107]. Additionally, even tissue-specific or inducible Cre drivers can have leaky or basal activity, causing unintentional gene modification outside the intended spatial or temporal window. This can complicate experimental interpretation, especially in tightly regulated biological processes. Furthermore, the system’s reliance on pre-inserted loxP sites limits its use to predefined genomic loci, making it unsuitable for de novo targeting of genes or large-scale genomic studies. This contrasts with technologies like CRISPR/Cas9, which allow direct targeting of nearly any sequence in the genome without pre-engineering. The system of CRISPR/Cas9 consists of an RNA sequence-guided Cas9 nuclease that introduces double-strand breaks at specific genomic locations. These breaks are repaired by cellular DNA repair mechanisms such as non-homologous end joining (NHEJ) or homology-directed repair (HDR), allowing precise gene knockouts or insertions. CRISPR/Cas9 is faster and more flexible than traditional methods, allowing multi-gene editing and rapid model development [104]. Unlike traditional Cre-loxP models, which require preinstalled loxP sites and extensive breeding for conditional knockouts, the CRISPR-based approach allows direct somatic editing of genes in immunocompetent mice. Su et al. [108], utilized the CRISPR/Cas9 method to generate autochthonous murine sarcoma models with a high tumor mutation burden, specifically targeting the Trp53 gene. By combining CRISPR/Cas9 with the chemical carcinogen 3-methylcholanthrene (MCA), sarcomas were induced in the gastrocnemius muscle by intramuscular injection of an adenovirus expressing Cas9 and a single guide RNA (sgRNA) targeting Trp53. Su et al. investigated the combined effects of radiotherapy (RT) and a Toll-like receptor 9 (TLR9) agonist (CpG) on sarcoma progression. After the tumors had reached a specified size, the mice were treated with Single doses of 20 Gy RT and CpG, either alone or in combination. The immune response to these treatments was characterised using bulk RNA-Seq, single-cell RNA-Seq and mass cytometry (CyTOF) to assess tumour growth delay, immune infiltration and CD8 + T-cell activation. or growth delay as the combination of CpG and RT significantly delayed tumour growth compared to either treatment alone, with a significant increase in time to tumour quintupling. CpG + RT-treated tumours showed increased infiltration of activated, proliferating CD8 + T cells expressing markers such as granzyme B and IFN-γ, critical for the antitumour response. The therapy enhanced antigen presentation pathways on myeloid cells, suggesting that the combination treatment altered the tumour microenvironment to promote T-cell activation. Notably. tumours in mice lacking functional CD8 + T cells or TLR9 did not respond to CpG + RT, confirming the dependence on these components for therapeutic efficacy. The combination of CRISPR/Cas9 and MCA induced sarcomas with high mutational burdens, better reflecting the genetic heterogeneity seen in human sarcomas.

The combination of these two powerful methods to create advanced GEMMs for sarcoma research has been studied [109], with a particular focus on undifferentiated pleomorphic sarcoma (UPS) and malignant peripheral nerve sheath tumours (MPNSTs). By integrating Cre-loxP and CRISPR-Cas9, the researchers achieved precise and efficient genetic manipulation to replicate human sarcoma pathologies in mice. The study used Cre-loxP technology to conditionally activate the oncogene Kras and delete the tumour suppressor Trp53. This was achieved by delivering Cre recombinase via adenoviral vectors to specific tissues. This method allowed highly controlled tumour initiation and mimicked the progression of human UPS. To increase the flexibility of model generation, the researchers also used CRISPR-Cas9 technology. They introduced Cas9 and guide RNAs (gRNAs) targeting Trp53 and Nf1 into mouse tissues using adenoviral vectors and in vivo electroporation. Combining technologies allowed the simultaneous disruption of multiple genes, accelerating tumour formation and enabling the study of complex genetic interactions. The models developed using this dual approach showed histological and molecular features that closely resembled human sarcomas. CRISPR-Cas9 proved particularly useful for introducing multiple mutations in a single step, which is challenging with traditional Cre-loxP models alone. The study also showed that CRISPR models can be generated more quickly and flexibly, making them ideal for studying rare sarcoma subtypes or testing multiple genetic hypotheses. This integration of Cre-loxP and CRISPR-Cas9 provides a robust framework for sarcoma modelling, combining the precision of conditional gene activation/deletion with the speed and versatility of genome editing. The study highlights the potential of these methods to advance sarcoma research and improve the development of targeted therapies.

## Conclusions

The primary advantage of GEMMs is their ability to faithfully mimic the full progression of tumour development in an immunocompetent and physiologically relevant microenvironment [110]. Unlike transplantation-based models, GEMMs allow researchers to study how specific genetic alterations, such as activation of oncogenes or loss of tumour suppressors, drive sarcomagenesis from its earliest stages to metastasis. For example, Cre-loxP technology allows precise spatial and temporal control of mutations such as Trp53 deletion and Kras activation, which can mimic the genetic and biological behaviour of human sarcomas such as undifferentiated pleomorphic sarcomas [111, 112]. These models are excellent at capturing the dynamic tumour-host interactions, including immune responses and microenvironmental changes, critical for studying sarcoma biology. GEMMs can reflect the influence of genetic drivers unique to sarcoma subtypes. For example, models driven by EWS-FLI1 expression accurately mimic Ewing sarcoma [113], while SS18-SSX2-driven models mimic synovial sarcoma [114], allowing detailed exploration of the biology and therapeutic vulnerabilities of these fusion-driven sarcomas. Another advantage is the immunocompetent nature of GEMMs, which facilitates the study of tumour-immune interactions and responses to immunotherapy [115]. This is particularly important for sarcomas, which are often considered immunologically ‘cold’ tumours [66]. GEMMs have shown how loss of tumour suppressor genes, such as Rb and Trp53, can influence immune evasion and the recruitment of immunosuppressive cells, providing insights into potential immunotherapeutic strategies [116]. These models are particularly valuable for testing immune checkpoint inhibitors, tumour vaccines or oncolytic viruses in a controlled, immunocompetent environment. In addition, GEMMs provide a platform for studying the synergistic effects of genetic mutations, as seen in CRISPR-enhanced GEMMs that allow for multi-gene modifications [117]. For example, models combining Nf1 and Trp53 deletions have been instrumental in studying malignant peripheral nerve sheath tumours (MPNSTs), revealing the interplay of these mutations in tumour progression and response to therapy [118]. GEMMs also enable longitudinal studies to understand the mechanisms of therapy resistance and tumour recurrence, which are critical to improving sarcoma treatment outcomes. Finally, GEMMs are essential for the preclinical testing of targeted therapies, radiotherapy and chemotherapy. Their ability to recapitulate human sarcoma biology underpins their utility in new drugs evaluation or therapeutic combinations, accelerating the translation of preclinical findings into clinical trials. The integration of advanced tools such as CRISPR-Cas9 has further streamlined the generation of GEMMs, enabling faster and more accurate modelling of rare or genetically complex sarcoma subtypes.

The main limitation of GEMMs is the significant time, cost and technical expertise required to develop and maintain them. Generating a single GEMM involves complex breeding strategies, often requiring multiple generations to achieve the desired genotype. This process can take months to years, delaying research timelines and increasing costs. In addition, the recombination efficiency of Cre-loxP systems can be variable, resulting in incomplete or mosaic gene modifications that complicate data interpretation. Even tissue-specific or inducible Cre drivers can exhibit leaky expression, causing unintended genetic changes that can confound experimental results. GEMMs often fail to fully capture the genetic heterogeneity observed in human sarcomas, particularly those with complex karyotypes such as osteosarcomas and dedifferentiated liposarcomas. While models such as Trp53 and Rb knockout mice recapitulate some aspects of these sarcomas [99], they cannot capture the extensive chromosomal instability or the full spectrum of mutations seen in human tumours. This limits the translatability of findings from GEMMs to the clinic. CRISPR-based GEMMs, while faster to develop, have their limitations. Off-target effects can introduce unintended mutations, and the high mutational burden often generated in these models may not accurately represent the stepwise progression of human sarcomas. In addition, while GEMMs are invaluable for studying tumour initiation and progression, they are less effective for modelling metastasis, as many GEMMs fail to replicate the specific metastatic patterns seen in human sarcomas, such as lung metastasis in osteosarcoma [119]. Finally, despite their immunocompetence, GEMMs still exhibit species-specific differences in immune responses that may not fully translate to human biology. For example, mouse immune systems differ in cytokine expression profiles, immune cell subsets and responses to immunotherapies, potentially confounding results when testing immune-based treatments.

## Key References

- Freeland J, Muñoz M, O’Donnell E III, et al. (2024) Genetic Screen in a Preclinical Model of Sarcoma Development Defines Drivers and Therapeutic Vulnerabilities. Clin Cancer Res 30:4957–4973.
  - This reference is of importance as it defined novel genetic drivers (YAP1, KRAS) and vulnerabilities in high-grade sarcoma models, linking specific oncogenic programmes to sarcoma subtypes and revealing oxidative phosphorylation as a potential therapeutic target. Outstanding for integrating TCGA data with functional modelling.
- Su C, Kent CL, Pierpoint M, et al. Enhancing radiotherapy response via intratumoral injection of a TLR9 agonist in autochthonous murine sarcomas. JCI Insight 9:e178767.
  - The reference is of outstanding importance as it presents developed CRISPR/Cas9-based murine sarcoma models with high tumour mutational burden by combining Trp53 editing and chemical carcinogenesis. Showed that radiotherapy combined with a TLR9 agonist enhances CD8 + T cell activation and delays tumour growth.
- Ozenberger BB, Li L, Wilson ER, Lazar AJ, Barrott JJ, Jones KB (2023) EWSR1::ATF1 Orchestrates the Clear Cell Sarcoma Transcriptome in Human Tumors and a Mouse Genetic Model. Cancers 15:5750.
  - This reference is of importance as it establishes a GEMM of clear cell sarcoma driven by the *EWSR1::ATF1* fusion, demonstrating that these models can faithfully reproduce both the histological and transcriptomic features of rare fusion-driven sarcomas.

## Data Availability

No datasets were generated or analysed during the current study.

## Declarations

### Transparency Statement

The lead author (AC) affirms that this manuscript is an honest, accurate, and transparent account of the study being reported; that no important aspects of the study have been omitted; and that any discrepancies from the study as planned (and, if relevant, registered) have been explained.
